# Unveiling wearables: exploring the global landscape of biometric applications and vital signs and behavioral impact

**DOI:** 10.1186/s13040-024-00368-y

**Published:** 2024-06-11

**Authors:** Carolina Del-Valle-Soto, Ramon A. Briseño, Leonardo J. Valdivia, Juan Arturo Nolazco-Flores

**Affiliations:** 1 Facultad de Ingeniería, Universidad Panamericana, Álvaro del Portillo 49, Zapopan, 45010 Jalisco Mexico; 2 Centro Universitario de Ciencias Económico Administrativas, Universidad de Guadalajara, Zapopan, 45180 Jalisco Mexico; 3 School of Engineering and Science, Tecnólogico de Monterrey, Monterrey, 64849 Nuevo Leon Mexico

**Keywords:** Behavior change techniques, Biometrics, IoT, Robotics for healthcare, Vital signs, Wearables

## Abstract

The development of neuroscientific techniques enabling the recording of brain and peripheral nervous system activity has fueled research in cognitive science. Recent technological advancements offer new possibilities for inducing behavioral change, particularly through cost-effective Internet-based interventions. However, limitations in laboratory equipment volume have hindered the generalization of results to real-life contexts. The advent of Internet of Things (IoT) devices, such as wearables, equipped with sensors and microchips, has ushered in a new era in behavior change techniques. Wearables, including smartwatches, electronic tattoos, and more, are poised for massive adoption, with an expected annual growth rate of 55% over the next five years. These devices enable personalized instructions, leading to increased productivity and efficiency, particularly in industrial production. Additionally, the healthcare sector has seen a significant demand for wearables, with over 80% of global consumers willing to use them for health monitoring. This research explores the primary biometric applications of wearables and their impact on users’ well-being, focusing on the integration of behavior change techniques facilitated by IoT devices. Wearables have revolutionized health monitoring by providing real-time feedback, personalized interventions, and gamification. They encourage positive behavior changes by delivering immediate feedback, tailored recommendations, and gamified experiences, leading to sustained improvements in health. Furthermore, wearables seamlessly integrate with digital platforms, enhancing their impact through social support and connectivity. However, privacy and data security concerns must be addressed to maintain users’ trust. As technology continues to advance, the refinement of IoT devices’ design and functionality is crucial for promoting behavior change and improving health outcomes. This study aims to investigate the effects of behavior change techniques facilitated by wearables on individuals’ health outcomes and the role of wearables in promoting a healthier lifestyle.

**Supplementary Information:**

The online version contains supplementary material available at 10.1186/s13040-024-00368-y.

## Introduction

The development of neuroscientific techniques that allow recording the physiological activity of the brain and the peripheral nervous system have driven research in cognitive science in recent decades. The technological age gives us new possibilities to induce behavioral change. The main advantage attributed to it is its good cost-effectiveness in people’s daily activities. The most effective Internet-based interventions to change behavior are based primarily on theoretical principles of the theory of planned behavior and use a series of techniques [1]. Until recently, the volume of equipment required to use these types of techniques restricted their use in the laboratory environment. This has been a limitation for the generalization of the results to real-life contexts. The available resources allow this information to be taken into people’s daily lives. The noteworthy point is that many of these resources are smart devices with sensors, microchips, and specific software that can connect to the Internet to exchange data with minimal human intervention, known as the Internet of Things (IoT) [2]. These devices connected to the network can be found in all kinds of objects: smart watches, electronic tattoos, refrigerators, air quality meters, traffic lights, drones, or heavy machinery for industry.

But what is the role of wearables? We have to see what is happening in the consumer market where predictions indicate that “the new mass phenomenon” is concentrated in wearables, whether these are active (capable of incorporating new services) or passive (designed for a single set of services). The latest projections indicate an annual growth rate of 55% over the next five years [3], which would mean an invasion of these devices in our daily lives. These products result from the technological evolution achieved with the miniaturization of electronic components, the development of communication protocols, geolocation, and data management software. Wearable technology represents a new stage in the evolution of the mobile device industry. If the first phase was characterized by social communication, that is, the possibility of communicating instantly, now it is moving towards a more personal environment [4]. Wearables began to be well known since they can measure a person’s daily activities to lead a healthier life, helping to carry out an analysis of such simple activities that are carried out daily. These devices have a microprocessor to perform certain functions, a battery, and a communication system with the user [5]. Wearables collect information and transform it to be able to show it to the user in the corresponding application and achieve follow-up. Wireless body sensor systems are needed to help people track what they want and provide data on their fitness. Large numbers of sales come from products aimed at the final consumer, but the applications of wearables are not limited to this market. Experts point out that the main opportunities are in industrial production. More and more companies have digitized plants and rely on wearables to integrate the latest IoT developments [6]. Items such as bracelets, watches or glasses, helmets and boots with sensors, smart vests and gloves are beginning to be incorporated. Nowadays, the use paradigm is the connected employee. These wearables give personalized instructions to each professional and, as a consequence, there is an increase in productivity and efficiency. The wearable market is booming due to its growing demand. There is a massive increase in the supply of these smart devices, especially in the field related to health. More than 80% of global consumers are willing to use this technology to monitor their vital signs and physical activity, among other uses [7]. This work aims to highlight the main biometric applications of people when using wearables. To understand the users’ primary needs, we consider people from the five continents (separating North America and Latin America). In addition, we establish relations of comfort, way of life, and health care associated with the way of life of people with and without wearables.

Behavior Change Techniques (BCTs) encouraged by the use of technology, particularly IoT devices such as wearables, have shown a significant impact on people’s health. The integration of computing capabilities with everyday tasks and activities has facilitated the collection and real-time monitoring of health-related information, enabling individuals to make informed decisions and take proactive steps towards improving their well-being [8]. This research aims to explore the effects of BCTs facilitated by IoT devices on behavior change and their subsequent impact on individuals’ health outcomes.

The utilization of wearables and other IoT devices in promoting behavior change has revolutionized the field of health monitoring and management. These devices, equipped with sensors and microchips, enable the seamless collection of physiological data, such as heart rate, sleep patterns, and physical activity levels [9]. By providing users with personalized insights into their health metrics, wearables empower individuals to track their progress, set goals, and modify their behavior accordingly. One of the primary advantages of IoT devices and wearables in facilitating behavior change is their ability to provide real-time feedback and personalized interventions. Through continuous monitoring and data analysis, these devices can deliver immediate feedback and tailored recommendations to users, prompting them to engage in healthier behaviors and make positive lifestyle choices [10]. For instance, wearables can send reminders to take breaks from sedentary activities, encourage regular physical exercise, or provide prompts for mindful eating.

The gamification of health-related activities through IoT devices, as demonstrated by Cho et al. [11], effectively promotes behavior change by engaging users with game-like elements, including challenges, rewards, and progress tracking. This gamified approach enhances adherence to healthy habits, fostering a positive reinforcement loop that encourages sustained behavior change. Moreover, IoT devices, as described by Xu et al. [12], seamlessly integrate with digital platforms like mobile apps and online communities, enhancing the impact of behavior change techniques. They allow users to access comprehensive health data and connect with support networks, such as online communities and peer networks. However, the use of IoT devices for behavior change raises privacy and data security concerns, as highlighted by Lansink et al. [13], due to constant data collection and transmission. Ensuring robust data protection measures and user consent is essential to maintaining trust and engagement. Despite these challenges, IoT devices, particularly wearables, have transformed behavior change techniques, leveraging real-time feedback, personalized interventions, gamification, and social support to empower individuals to adopt healthier lifestyles and make informed decisions about their well-being. To maximize their potential in promoting positive behavior change and improving health, ongoing advancements in device design and functionality should address these privacy concerns as technology continues to evolve.

## Related work

Wearables record what we see, our heart rate, our breathing rate, the hours we sleep, and even our feeling of well-being or stress. They also reinforce good eating and health habits. These products result from the technological evolution achieved with the miniaturization of electronic components, the development of communication protocols, geolocation, and data management software. Wearables are used to monitor the vital signs and biometrics of people. Biometrics is a method of recognizing people based on their physiological or behavioral characteristics. There are two main types of biometrics: physiological and behavioral. The physiological type takes characteristic features of our body as a reference to turn them into parameters or indicators. The behavioral type uses other aspects of a specific action, such as writing, walking, or sleeping, among other types of the proposal they make in the work cited in [14].

Wearables have gained popularity for monitoring various aspects of life, including health and well-being. They encompass a wide range of applications, from fitness tracking to healthcare and industrial uses [15]. Biometrics, both physiological and behavioral, play a significant role in enhancing wearables’ functionality. In the realm of wearable technology, numerous researchers have been actively contributing to the burgeoning field, exploring various dimensions and applications. Neethirajan’s paper [16] serves as a valuable reference, shedding light on the multifaceted role of wearables in monitoring diverse aspects of life, from health and well-being to applications in fitness tracking, healthcare, and industrial contexts. This review paper has sparked extensive discussions and investigations among scholars. Additionally, other noteworthy studies, such as Din et al.’s research on wearable biometric sensors for continuous health monitoring [17] and Low’s exploration of behavioral biometrics in wearables for authentication [18], have significantly enriched our understanding of the integration of biometrics, both physiological and behavioral, in enhancing the functionality and utility of wearables.

Physiological biometrics, such as heart rate and temperature monitoring, offer valuable insights into users’ health. Behavioral biometrics, like gait analysis or signature recognition, provide unique identifiers for security and authentication [19]. Traditional biometric modalities, like fingerprint and facial recognition, have also found applications in wearables.

The quality and accuracy of sensors integrated into wearables are crucial for their reliability and usefulness. Advances in miniaturization have allowed manufacturers to create smaller, more precise sensors, contributing to the popularity of wearables [20]. These devices are used in various fields, including healthcare, sports, entertainment, and industry. Mengüç et al.’s seminal work in [21] has been instrumental in emphasizing the significance of sensor quality and accuracy in the context of wearables, acting as a catalyst for further investigations. Researchers across diverse domains have been actively engaged in advancing the miniaturization and performance of these sensors to meet the burgeoning demands of wearable applications. For instance, Hao and colleagues have made notable contributions to the field through their exploration of advanced sensor technologies for healthcare monitoring [22], while Vital et al. have delved into sensor innovations tailored for sports and fitness wearables [23]. Furthermore, in the realm of entertainment and gaming wearables, the research by Athota et al. has pushed the boundaries of sensor integration and immersive user experiences [24].

Recent advancements in neuroscientific techniques have enabled the recording of physiological activity in both the brain and peripheral nervous system, driving significant research in cognitive science over the last few decades. The advent of the technological age has introduced new possibilities for inducing behavioral change [25], with a key advantage being its cost-effectiveness in integrating with people’s daily routines. Internet-based interventions designed to modify behavior draw heavily from theoretical principles of the theory of planned behavior and employ a range of behavior change techniques [26]. However, the use of such techniques was previously constrained to laboratory settings due to the volume of equipment required, limiting the generalizability of findings to real-life contexts.

The proliferation of smart devices equipped with sensors, microchips, and specialized software capable of internet connectivity, often referred to as the IoT [27], has overcome this limitation. These IoT-enabled devices have found applications across various domains, including smartwatches, electronic tattoos, air quality meters, and even heavy machinery in industrial settings.

While a substantial portion of wearables is targeted at the consumer market, their applications extend beyond to industrial production. Industries are increasingly relying on wearables to integrate the latest IoT advancements [28]. Products like sensor-embedded bracelets, watches, helmets, boots, smart vests, and gloves are gradually becoming part of the workforce’s standard equipment, fostering personalization and efficiency [29].

Research in [30] aims to underscore the primary biometric applications of wearables in monitoring people’s vital signs. This research endeavor seeks to illuminate the paramount role played by biometric applications in wearables, particularly their proficiency in monitoring individuals’ vital signs. Within this burgeoning field, several notable contributions have emerged. For instance, Muller and colleagues have conducted substantial work elucidating the potential of wearables in chronic disease management and preventive healthcare [31]. Simultaneously, the study by Elshafeey et al. has delved into the integration of artificial intelligence with wearables for more robust vital sign monitoring and health assessment [32]. Furthermore, the research conducted by Stark and co-authors has delved into the challenges and opportunities in applying wearables for remote patient monitoring in healthcare settings, emphasizing the need for further advancements in data security and reliability [33].

Amiri and colleagues have explored the efficacy of BCTs, delivered via mobile applications and wearables, in promoting physical activity and healthier lifestyles [34]. Additionally, the study by Trinidad and co-authors delves into the utilization of IoT-based wearables for behavior change interventions targeting specific health conditions, such as obesity and diabetes management, further emphasizing the potential of these technologies in personalized healthcare [35]. Furthermore, the work conducted by Teixeira et al. has investigated the role of wearable IoT devices in facilitating behavior change among elderly populations, highlighting their utility in promoting independence and health in aging individuals [15].

Wearables and other IoT devices have revolutionized health monitoring and management by seamlessly collecting physiological data such as heart rate, sleep patterns, and physical activity levels [9]. Real-time feedback and personalized interventions represent one of the primary advantages of IoT devices and wearables in promoting behavior change. Continuous monitoring and data analysis enable these devices to deliver immediate feedback and tailored recommendations, encouraging users to adopt healthier habits and make informed choices [10].

Gamification of health-related activities through IoT devices has emerged as an effective strategy for behavior change. By incorporating game-like elements such as challenges, rewards, and progress tracking, wearables foster engagement and motivation among users. This gamified approach enhances adherence to healthy behaviors and sustains behavior change over time [11].

Furthermore, IoT devices seamlessly integrate with digital platforms, including mobile applications and online communities, amplifying the impact of behavior change techniques. Wearables can communicate with smartphones and other connected devices, allowing users to access comprehensive health data and connect with support networks [12]. The availability of social support through online communities fosters accountability and encourages individuals to maintain health-focused behaviors.

However, it is crucial to acknowledge the potential challenges and limitations associated with IoT devices for behavior change. Privacy and data security concerns arise due to constant data collection and transmission. Robust data protection measures and user consent are essential to maintain trust and engagement in these devices [13]. As technology continues to evolve, addressing these concerns and refining device design and functionality are paramount to maximize the potential of IoT devices in promoting positive behavior change and improving overall health.

The integration of wearable technology and IoT devices has ushered in a new era of health monitoring and behavior change. These devices, equipped with sensors and microchips, empower individuals to take control of their health by providing real-time feedback, personalized interventions, and gamified experiences. While privacy concerns persist, the potential for improving well-being through these devices remains substantial, making them a pivotal tool in the realm of healthcare and behavior change.

### Biometrics applications

Sensors in the different devices collect and emit data constantly. These sensors can be classified into three main categories: motion sensors (convert mechanical movement into an electrical signal), physiological (use optical, electrical, acoustic, or thermal sensing components to measure vital parameters such as heart rate, temperature, blood pressure, or blood oxygen saturation, bioelectrical activity such as electrocardiography or electroencephalography) and biochemical (used to measure chemicals such as glucose, electrolytes) [36]. The application of biometrics is an increasingly common aspect of people’s motivation to buy wearable devices. Our passwords and access controls are becoming more sophisticated and evolving as the processing and amount of data we deal with daily evolves. Recent situations have revealed not only the vulnerability in the custody of our data today but also the risks of making malicious use of them. Data is the new currency. The new privacy requires new rules, passwords, and other levels of security. In this panorama, our body has become a unique identifier. We have distinctive aspects that are not repeated in any other person. The iris, the geometry of the hand, or body odor can become biometric (physiological) identifiers. In addition, the way of writing, walking, or recognizing our signature as unique are identifiers of a behavioral type [37]. Many traditional biometric modalities, such as fingerprint, face, iris, and voice recognition, can now be applied to body-worn device formats. Fingerprint authentication is already supported by smartphones and could be applied to watches and wristbands using fingerprint swipe sensors. Similarly, because many wearables already incorporate cameras, facial recognition represents the logical choice for smart glasses and body-worn cameras [38].

Recent studies, such as cited in [39], have shown that wearable devices equipped with real-time feedback mechanisms can effectively move individuals from contemplation to preparation and action by setting personalized goals and providing feedback on achievements. These studies highlight the dynamic capabilities of wearables to adjust their feedback and motivational strategies based on the user’s current readiness to change, thus supporting a more tailored and effective intervention approach.

Advanced analytics integrated into wearables can predict the user’s stage of change by analyzing behavioral patterns and physiological data. For instance, a study by Oyebode et al. [40] utilized machine learning techniques to analyze data from wearables to predict when individuals were likely to advance to the next stage of behavior change. This predictive capability allows for proactive adjustments to the behavior change strategies employed by the wearable. Longitudinal studies that track the progression through the stages of behavior change over time are essential to evaluate the effectiveness of wearables in supporting sustainable behavior change. For example, the work by Strath et al. [41] investigated the long-term impact of wearables on maintaining physical activity levels in previously inactive individuals, demonstrating sustained engagement and improved health outcomes over a 12-month period.

### Wearables uses

Wearables manufacturers are increasingly concerned with improving the accuracy of their products. This is causing a boost in the different categories of wearable devices. The quality of the integrated sensors is an essential factor in defining the reliability and usefulness of the product. The precision of these sensors is more significant every day, obtaining more precise measurements. The rapid advances in miniaturization allow manufacturers to integrate increasingly smaller sensors and thus reduce the size of the devices. One of the main characteristics of these devices is that they do not need to be switched on or off, so they do not stop fulfilling their function. Depending on the utility, sensors in wearables can be classified based on their use in consumer health, research, or healthcare. At the healthcare level, primary care professionals integrate wearables into clinical practice to monitor patients’ health, manage chronic diseases, diagnosis and treatment of diseases, and rehabilitation. Some examples of uses include accelerometers or motion sensors and atrial fibrillation detection sensors [36]. They can be used alone or with other parameters such as heart rate. In research, measurements from wearables can yield statistics for early model predictions based on people’s behavior or daily life. These complex algorithms can receive real-time feedback and estimate patterns to anticipate people’s needs or requirements. For example, suggesting routes with less traffic by knowing the destination in advance, proposing a warm and comfortable environment in the home before the person’s arrival, or suggesting food promotions or personalized stores to the consumer [42]. They can even be an extension of the user’s body or mind. The most common areas in which they are used are (a) Health, (b) Sports, (c) Welfare, (d) Entertainment, (e) Industrial, and (f) Military [43]. A Wearable is generally used to help improve the health and well-being of people, animals, or even plants. These categories can be grouped into an application-based classification of wearables, such as (1) Access control, (2) Online navigation control, (3) Presence control, (4) Fight against fraud, (5) Authentication, and (6) Payment method.

## Methodology

The methodology of this research work consists of two large parts. The first is a survey of the primary uses of wearables worldwide, the advantages and disadvantages found in their uses, and people’s appreciation (classified by continents) regarding their positive and negative aspects and degree of satisfaction. This activity was accomplished by describing the activities and perceptions of individuals using wearables for an entire week and a complete week without them, in order to annotate differences. The second part consists of the impact of behavioral change techniques on the principal vital signs of people. So, here we study each behavioral change technique separately in each person through activities that represent it, considering not using wearables from any other technique. In this way, we observe the impact on vital signs such as Breathing frequency (BF), Deep sleep (DS), Heart rate (HR), Oxygen saturation (OS), REM sleep (REMS), and Temperature (T).

To evaluate the impact of these techniques, an experiment was conducted with users wearing their usual wearables for one week, followed by a week without wearables. Users’ perceptions of behavioral change varied, with some techniques receiving positive feedback while others did not. The number of indicators met per day for each technique also decreased during the week without wearables.

Results from the experiment align with users’ satisfaction levels, with goal setting and personalization being highly appreciated. Additionally, the impact on vital signs was analyzed, revealing significant differences among techniques. Goal setting emerged as the most effective technique in promoting well-being, both in user perception and vital sign impact.

The inclusion of the transtheoretical model in studies concerning behavior change facilitated by wearable devices is crucial due to its comprehensive framework that segments behavior change into distinct stages. This model aids in understanding the progression through stages of precontemplation, contemplation, preparation, action, and maintenance, which are essential for realizing long-term behavior changes.

By assessing which stage of behavior change an individual is in, wearables can be tailored to provide stage-specific interventions. For example, for someone in the contemplation stage, wearables could provide motivational messages and reminders about the benefits of change, while for those in the action stage, more intense feedback on progress and tips to overcome challenges could be offered.

Wearable technologies can be effectively utilized to support individuals through these stages. For instance, in the precontemplation stage, where an individual may not yet recognize the need for change, wearables can provide data that highlights health risks or behaviors that may motivate recognition of the need for change. In the contemplation and preparation stages, wearables can offer personalized feedback and goal-setting tools to help users plan for change.

In the action and maintenance stages, wearables can play a critical role by offering continuous support and feedback. This can include tracking progress towards goals, providing motivational messages, and even offering rewards for achieving certain milestones. For example, a wearable could remind an individual to engage in physical activity regularly or monitor dietary habits to support weight management goals.

Considering a sample of 30 people for each continent (we separate America into North America and Latin America), we interviewed these people about their perception of wearables, quantity, and prominent use. The results are shown in Table 1, where the most significant number of wearables per person is found in North America, Europe, and Asia. We classify the main applications of wearables from the biometric point of view and find that people in Latin America prefer to have more Smart Watches with 93%. We observe applications related to Fitness trackers and health gadgets, Smart devices for sports, Virtual reality goggles, and Hearables, which predominate in Oceania with 91%, 92%, 25%, and 62%, respectively. Smart jewelry applications are accounted for mainly in North America with 31%. Smart clothes and implantable device applications predominate in Asia with 32% and 25%, respectively. Regarding the type of biometric applications, the people who use both types (Physiological and Behavioral) with equal importance belong to Latin America, Europe, and Asia. The rest of the people predominantly use the Behavioral type.

**Table 1:** Application and average biometric type of wearables by continent

People for continent	Average QTY of wearables	Application	Biometric type
North America	8	- Smart Watches 90% - Fitness trackers and health gadgets 88% - Smart devices for sports 82% - Smart jewelry 31% - Smart clothes 22% - Virtual reality goggles 19% - Hearables 56% - Implantable devices 18%	Behavioral
Latin America	5	- Smart Watches 93% - Fitness trackers and health gadgets 80% - Smart devices for sports 62% - Smart jewelry 11% - Smart clothes 9% - Virtual reality goggles 18% - Hearables 41% - Implantable devices 2%	Physiological and Behavioral
Europe	7	- Smart Watches 95% - Fitness trackers and health gadgets 80% - Smart devices for sports 86% - Smart jewelry 20% - Smart clothes 25% - Virtual reality goggles 21% - Hearables 49% - Implantable devices 10%	Physiological and Behavioral
Asia	6	- Smart Watches 80% - Fitness trackers and health gadgets 85% - Smart devices for sports 88% - Smart jewelry 35% - Smart clothes 30% - Virtual reality goggles 34% - Hearables 60% - Implantable devices 25%	Physiological and Behavioral
Africa	4	- Smart Watches 70% - Fitness trackers and health gadgets 61% - Smart devices for sports 80% - Smart jewelry 12% - Smart clothes 5% - Virtual reality goggles 5% - Hearables 11% - Implantable devices 9%	Behavioral
Oceania	5	Smart Watches 95% - Fitness trackers and health gadgets 91% - Smart devices for sports 92% - Smart jewelry 21% - Smart clothes 20% - Virtual reality goggles 25% - Hearables 62% - Implantable devices 15%	Behavioral

With the use of different types of wearables, we can analyze more effectively the utility that an individual could derive from each device. Through this utility assessment, we can further analyze people’s preferred activities based on continents around the world. By understanding these activities, we can delve deeper into examining the behavioral change techniques that individuals may develop.

**Fig. 1:**
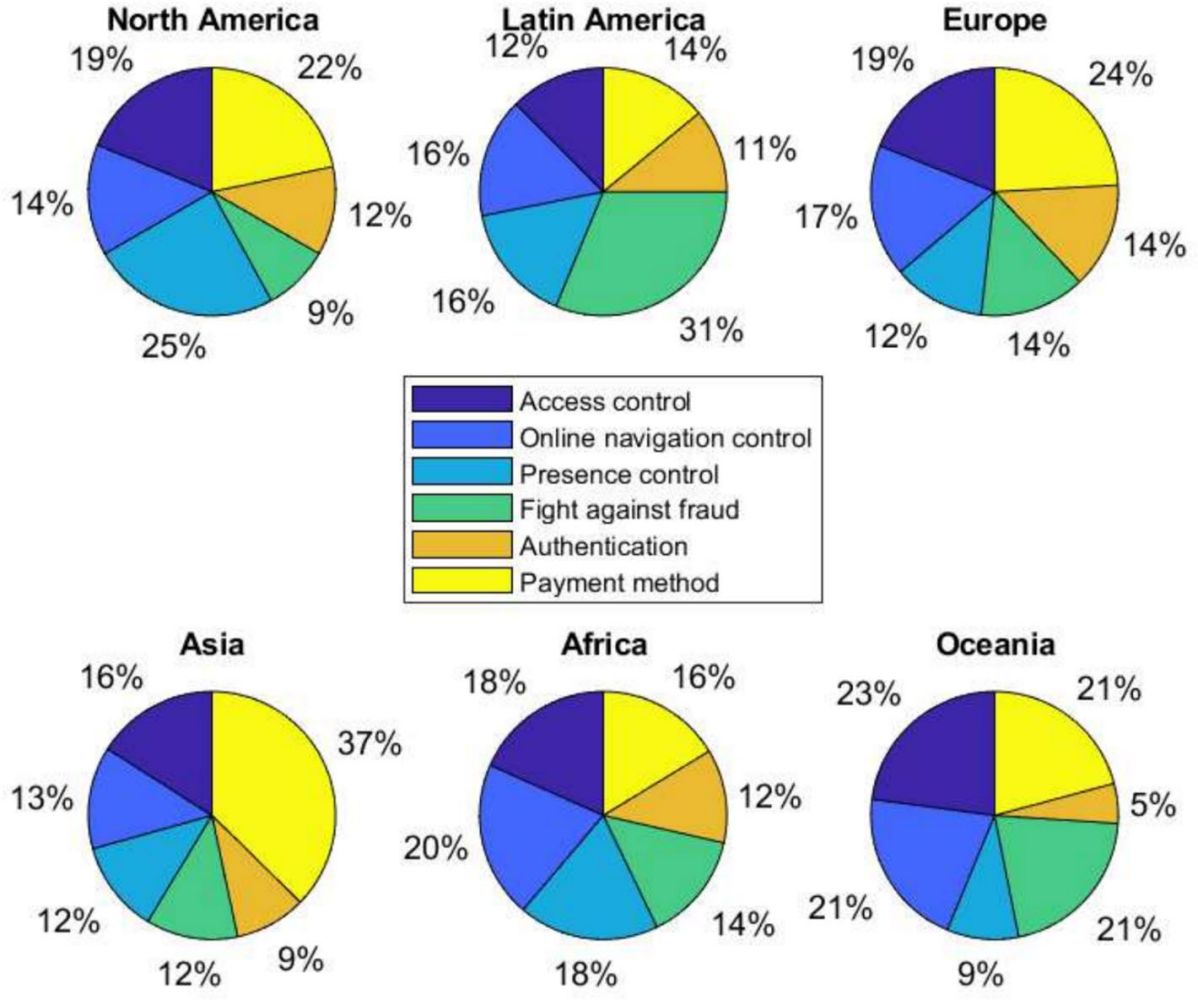
Main uses of wearables from biometrics in the five continents

Among the main uses, we can give to wearables or areas in which they are used. We can highlight sports to measure our sports activity, steps and distances traveled, level of calories burned during exercise, and track position. In health, to monitor a person’s blood pressure, heart rate, temperature, and other vital signs. Our daily tasks include answering calls, receiving messages, checking email, alarms, and customizing our favorite music. However, from a biometric point of view, wearables are also helpful in identifying and associating profiles, authenticating people, detecting fraud, and recognizing behavior patterns. Figure 1 collects information from 30 people for each continent and quantifies, as a percentage, the main uses that people have in each area of the planet. For example, in Latin America, most wearables are used to fight against fraud; in Europe and Asia, the most important use is for payment. It is interesting to find that in Oceania, the most significant use is given to Access control. In North America, the primary use is for Online navigation control. In Africa, the uses are distributed similarly, with Online navigation control standing out. These uses may be related to people’s daily activities and social, economic, and cultural environments. There are areas where the automation of the environment has become the priority of people where they live. This generates fear regarding the control of the applications and their security. This situation also responds to people’s need to control their environment and comfort scale in a pyramid of priorities. All this in a single device with remote control of everything.

**Fig. 2:**
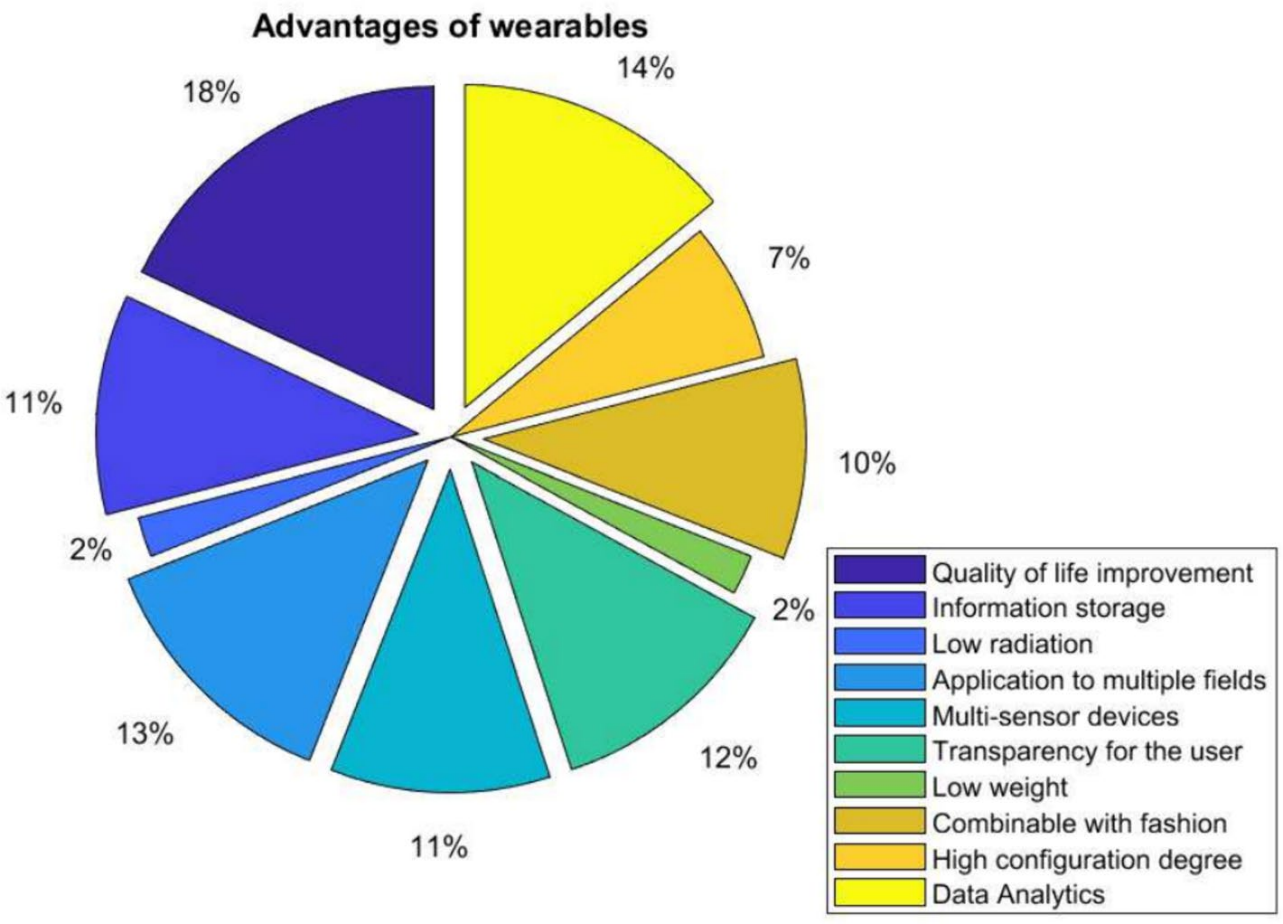
Main advantages of wearables based on user opinion

Figure 2 shows the main advantages of wearing wearables according to the opinion of 180 people (equivalent to 30 people per continent, dividing North America and Latin America). The most relevant advantage people find is Quality of life improvement followed by Data Analytics and Transparency for the user. The less relevant advantages are Low radiation and Low weight. People’s perspective on the best comfort in quality of life and all the decisions they can make based on all the data they get from their bodies daily is interesting.

Selection Criteria and Demographic Distribution: Participants were selected based on their willingness and availability to engage in a longitudinal study involving wearables. The demographic distribution aimed at a balanced representation of age and gender to mitigate bias in behavior change response due to these variables. Each continent contributed equally to the pool with an equitable mix of urban dwellers, ensuring varied environmental and lifestyle contexts were considered in the study.

Experience with Wearables: Prior to the study, participants were surveyed to determine their familiarity and prior usage of wearable technology. This was a crucial factor as previous exposure to such technology could influence the responsiveness to behavior change interventions facilitated by wearables. The survey revealed a mix of experienced and novice wearable users, allowing the study to further analyze how familiarity with wearables impacts the effectiveness of behavior change techniques.

Impact on Behavior Change: The methodology was designed to track and analyze the impact of wearables on inducing behavior changes across various setups and conditions. The study specifically looked at how these devices influenced daily routines, health monitoring, and proactive health management, with a focus on both immediate and long-term behavior changes.

**Fig. 3:**
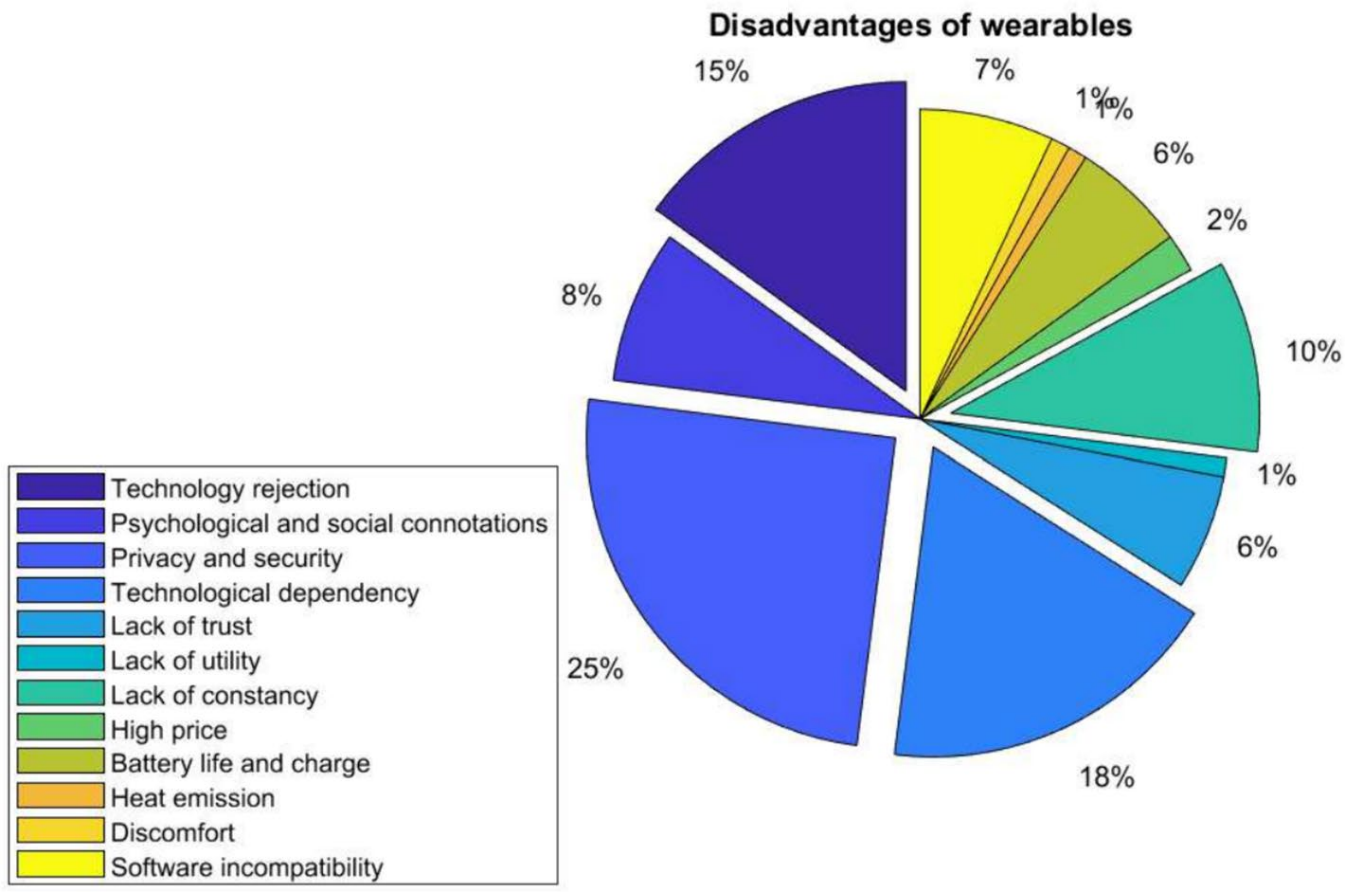
Main disadvantages of wearables based on user opinion

Figure 3 shows the main disadvantages encountered by users. The main disadvantage found is Privacy and security. It may be because privacy is based on whether or not the person can decide whether or not the device records a location, for example. Moreover, security is based on the fact that only who authorizes the person has access to the information. Another major drawback is Technological dependency. People believe that the social impact of technology today is breaking strong ties and creating needs that never existed before. The less relevant disadvantages are Lack of utility and High price. This means that people generally find wearables very useful and do not consider them expensive or only for one sphere of society. Security concerns regarding biometric login information stored in the cloud are the most significant obstacle to incorporating biometrics into wearable technology. Technology, format, and cost were not generally seen as impediments.

Wearables offer several advantages, including real-time data collection, personalized interventions, and gamification strategies that enhance user engagement and motivation. The seamless integration of wearables with digital platforms facilitates comprehensive tracking and analysis of BCTs, enabling researchers to assess their effectiveness in promoting behavior change. However, it is essential to acknowledge potential disadvantages, such as privacy and data security concerns, which require careful consideration to ensure user trust and data integrity. As researchers continue to explore the multifaceted roles of wearables in BCT analysis, a comprehensive understanding of their uses and associated benefits and drawbacks is crucial for advancing the field of behavior change science.

Behavioral change can be fostered through complementary methods of communication motivated by connected devices all the time. For example, weight loss is made more effective through reminders that enhance wearables, emerging as excellent allies for behavioral interventions. The uses described in the previous section allow a more detailed classification of wearable applications. Table 2 details five representative activities for each behavior change technique. These activities are the most common in the daily use of wearables, according to the 180 people surveyed, and have been classified as the most studied behavioral techniques in the literature [36]. Each activity is exemplified using five representative indicators chosen by the people surveyed as the most common.

**Table 2:** Detail of activities by behavior change technique

Behavior change techniques	Activities
Induction	1. Phone reminders, 2. Check in places to have a record of favorites, 3. Reminder for shopping list, 4. Smart alert system, 5. Reminders to improve sleep behavior.
Self-appraisal	1. Daily activity notes, 2. Daily food notes, 3. Sleep activity log, 4. Monitoring of periodic physiological functions, 5. Analysis of physical complexion.
Goal setting	1. Mark number of steps, 2. Time for shorter routes, 3. Monitoring of healthy lifestyles, 4. Total burned calories, 5. Ideal weight monitoring.
Gamification	1. Earn points for every n steps, 2. Fitness Monitoring Cycle, 3. Collective challenges without borders, 4. Systems that include immersive video games, 5. Advanced dialog interfaces.
Personalization	1. Send personalized messages, 2. Position and activity data may recommend fluid intake, 3. Position and search data to send advertising and promotions, 4. Social rank expectation and confirmation, 5. Classification of profiles to engage in personal communication.
Behavior self-monitoring	1. Provide graphs and messages with feedback tailored to progress, 2. Reinforcement learning, 3. Measurement of levels of concentration, stress and attention, 4. Participatory learning, 5. Portable data monitoring.
Social support	1. Social network to share experiences and strategies, 2. Help people with disabilities in real time, 3. Visit patterns for marketing quality, 4. Gesture control, 5. Interactive online community.
Provision of instructions	1. Detailed instructions of tasks that can be performed to reduce sedentary behavior, 2. Make shopping list, 3. Breathing exercises, 4. Auditory and visual route tracking, 5. Prioritizing the use of applications for productivity.

In this way, we exposed 30 randomly selected individuals from the same sample of 180 previously mentioned for the surveys, to the 8 behavior change techniques studied (Goal setting, Induction, Provision of instructions, Social support, Self-appraisal, Behavior self-monitoring, Gamification, and, Personalization). the experiment lasted 8 weeks, one week per technique. For each week, the samples used the wearables only in activities related to the corresponding technique. Additionally, during the experiment, we measured the people’s vital signs to assess the impact of behavior change techniques on them. Also, in this experiment, the samples fill out a survey with the level of satisfaction for the different activities experimented in each behavior change technique.

In order to find statistically significant differences in the vital signs measured between the different behavior change techniques, first, we verified whether these follow a normal distribution or not with the Shapiro-Wilk test (see Eq. 1).

1 $$\begin{aligned} W = \frac{{\left( \sum _{i=1}^{n} a_i x_{(i)}\right) ^2}}{{\sum _{i=1}^{n} (x_i - \bar{x})^2}} \end{aligned}$$

Where:

*W*: represents the Shapiro-Wilk test statistic.

*n*: denotes the sample size.

$x_i$: corresponds to the ordered values of the sample, i.e., the i-th smallest value to the i-th largest value.

$x_{(i)}$: refers to the expected standardized values for a sample of size *n* drawn from a normal distribution. These values are used as coefficients in the formula.

$\bar{x}$: stands for the sample mean.

$a_i$: are the calculated coefficients that depend on the sample size *n*. These coefficients are used to adjust the expected values $x_{(i)}$ and are employed in the computation of the test statistic *W*.

Second, if the measured vital signs followed a normal distribution it is necessary to use a parametric test like the repeated measures ANOVA (see Eq. 2) for each set of measurements per vital sign. Otherwise, if at least the measures of one vital sign of one behavior change technique do not follow a normal distribution, the set of measurements of that vital sign has to be analyzed using a Friedman test (see Eq. 3).The corresponding test (ANOVA or Friedman) checks if there are statistically significant differences in each set of measurements per vital sing across the behavior change techniques.

2 $$\begin{aligned} F = \frac{{MS_{\text {between}}}}{{MS_{\text {within}}}} \end{aligned}$$

where:

*F*: is the *F*-statistic of the ANOVA, which follows an *F*-distribution under the null hypothesis.

$MS_{\text {between}}$: is the mean square for differences between the group means (between-group variance).

$MS_{\text {within}}$: is the mean square for differences within the groups (within-group variance).

3 $$\begin{aligned} \chi ^2 = \frac{{12}}{{nk(n+1)}} \sum \limits _{j=1}^{k} R_j^2 - 3(n+1) \end{aligned}$$

Where:

$\chi ^2$: represents the Friedman test statistic.

*n*: is the number of observations (subjects).

*k*: is the number of groups.

$R_j$: is the sum of ranks of observations in group *j*.

After, If significant differences were found in the vital signs between the different behavior change techniques, post hoc tests are performed to identify which behavior change techniques have a positive and negative influence on the well-being of the persons. The post hoc tests can be parametric with a t-student test (see Eq. 4) or non-parametric with Wilcoxon test (see Eq. 5). Finally, we made a rank for the 8 behavior change techniques base on the well-being of the person’s vital signs.

4 $$\begin{aligned} t = \frac{{\bar{x}_1 - \bar{x}_2}}{{\sqrt{\frac{{s_1^2}}{{n_1}} + \frac{{s_2^2}}{{n_2}}}}} \end{aligned}$$

where:

$t$: is the t-value of the independent samples t-test.

$\bar{x}_1$ and $\bar{x}_2$: are the means of the two independent samples being compared.

$s_1^2$ and $s_2^2$: are the variances of the two samples.

$n_1$ and $n_2$: are the sizes of the two independent samples.

5 $$\begin{aligned} T = \sum \limits _{i=1}^{n} \text {sgn}(d_i) \times \min (|d_i|) \end{aligned}$$

where:

*T* is the Wilcoxon Signed-Rank Test statistic.

$d_i$ represents the paired differences between the observations.

$\text {sgn}(d_i)$ is the sign function, which returns 1 if $d_i$ is positive, -1 if $d_i$ is negative, and 0 if $d_i$ is zero.

$\min (|d_i|)$ is the smallest absolute value of the observed differences.

By other hand, with a satisfaction survey over the 30 samples of this experiment, we explore and plot the statistical description of the results with the aim of comparing them with the vital signs measures results.

## Implementation

There is a wide variety of behavioral change techniques to improve lifestyle. These techniques are used by devices to modify people’s behavior to improve their health or life. There are simple techniques, such as health advice through text messages, to more complex and innovative techniques, such as gamification and interventions that adapt to the psychological state of the user and their environment. Other examples would be goal setting, feedback on results and follow-up, self-assessment, or personalized messages. Some of the behavior change techniques are considered to be more effective on the health factor in question [44]. With the approach of the opinions of the sample of people worldwide, we propose an experiment with the same people based on the fact that they use their wearables one week typically and another week they no longer use any of them. All participants gave their informed consent.

### Results

Before commencing with a quantitative analysis of the impact of wearables on BCTs, we examined the level of satisfaction individuals experienced with the use they detected in wearables. This was done to ensure that they could feel at ease with the tests and genuinely use their device for some well-being purpose.

Table 3 shows an experiment conducted with the 180 people surveyed during this study. People will carry all the wearables they usually use for daily activities for a week. For another week, people are not going to use these wearables. We analyzed the behavioral change perception according to the techniques presented in Table 2 (positive, negative perception, and no effect). In addition, we studied the number of indicators met per day for each behavioral change technique. Each person performs a total of these indicators at the end of the day.

**Table 3:** Perception and use of activities by behavior change technique

Behavior change techniques	With wearables		No wearables	
	Behavior change	Number of indicators	Behavior change	Number of indicators
Induction	Positive	5	Negative	2
Self-appraisal	Positive	5	Not affected	4
Goal setting	Positive	5	Negative	2
Gamification	Positive	3	Negative	0
Personalization	Positive	5	Negative	2
Behavior self-monitoring	Positive	4	Negative	0
Social support	Not affected	3	Not affected	2
Provision of instructions	Not affected	3	Not affected	3

We found that users’ perception of behavioral change is positive with the use of wearables in techniques such as Induction, Goal setting, Gamification, Personalization, and Behavior self-monitoring. Also, we observe that two techniques are not perceived as positively dependent on wearables: Social support and Provision of instructions. Regarding the five most common indicators of each behavioral change technique, we noticed that in most of the techniques, the users reduce the potential of said technique by reducing the indicators by more than 50%. This leads us to think that people lose interest in activity monitoring for their health, well-being and/or productivity.

**Fig. 4:**
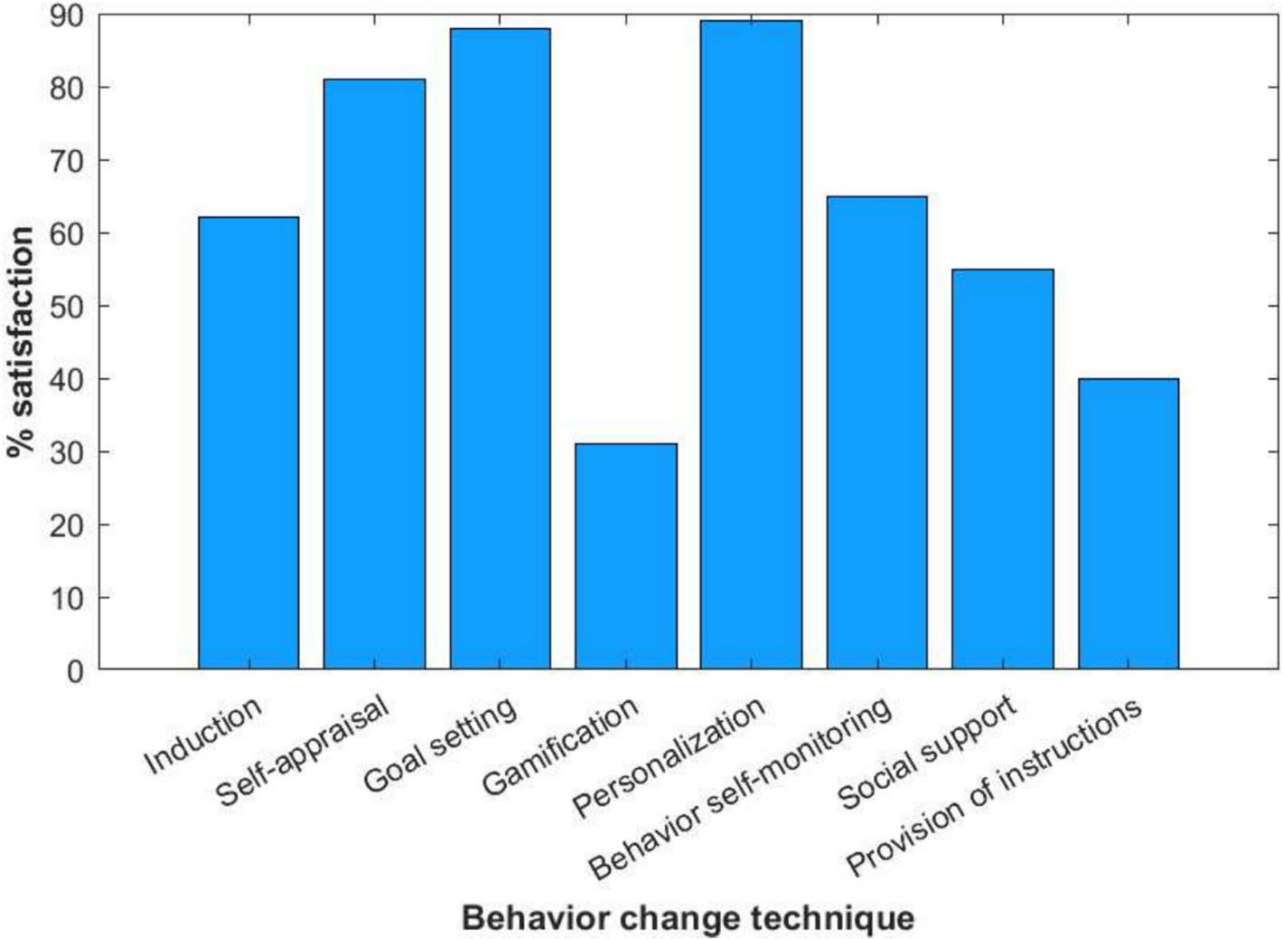
Percentage of satisfaction with behavioral change techniques with the use of wearables

Figure 4 shows the percentage of satisfaction with behavioral techniques perceived by people when using wearables. These devices have functionality for comfort, interaction, and validation of the body’s behavior since they calculate indicators of the user’s current state of health and recommend action plans to improve said indicators. Wearables provide immediate monitoring and feedback (interacting continuously with the user and with other devices). We evaluated the degree of perception of satisfaction of people from the point of view of behavioral techniques. So, we take an average of the percentage that the person considers that the use of wearables impacts that behavior. We found that techniques such as Self-appraisal, Goal setting, and Personalization are the most appreciated and with the most significant impact benefited by wearables.

Table 4 provides typical values for several key physiological parameters, which are crucial for monitoring an individual’s health status using wearable devices. These ranges serve as benchmarks to determine whether the readings taken by the wearables are within normal limits or if they deviate, potentially indicating an impact on the individual’s daily life. For example, Heart rate deviations from this range could indicate cardiovascular issues, stress, or other health concerns. Breathing frequency variations outside this range might suggest respiratory problems or changes in metabolic demand. Temperatures outside this range could indicate fever, infection, or other medical conditions. Oxygen saturation and its levels below this range may indicate hypoxemia or other pulmonary issues. REM sleep deviations from this range can affect cognitive function, mood, and overall health. Insufficient deep sleep can impact physical recovery, immune function, and memory consolidation. By consistently monitoring these metrics, wearable devices can alert users to potential health issues, allowing for timely interventions and adjustments to their daily activities to maintain optimal health [45].

**Table 4:** Vital signs metrics

Metric	Typical values
Heart rate (HR)	60 to 100 beats per min
Breathing frequency (BF)	12 to 18 breaths per min
Temperature (T)	97.8 ^∘^F to 99.1 ^∘^F (36.5 ^∘^C to 37.3 ^∘^C)
Oxygen saturation (OS)	95–100%
REM sleep (REMS)	between 20–25% of total sleep time
Deep sleep (DS)	between 10–20% of sleep time

With the experiment to analyze the impact of the behavior change technique, on the vital signs we found the following results:

In the Shapiro-Wilk normality tests of the set of vital sign samples, hypothesis H0 denotes that the data follow a normal distribution. If the p-value resulting from a Shapiro-Wilk test is lower than 0.05 the H0 is annulled with a 95% of confidence interval and consequently, the analyzed sample doesn’t follow a normal distribution.

**Table 5:** Results of Vital Signs Shapiro-Wilk Normality Test

Vital sign	Sig.	Vital sign	Sig.
BF_Behavior-self-monitoring	0.042	OS_Behavior-self-monitoring	0.001
BF_Gamification	0.187	OS_Gamification	0.271
BF_Goal-setting	0.332	OS_Goal-setting	0.012
BF_Induction	0.222	OS_Induction	0.003
BF_Personalization	0.166	OS_Personalization	0.002
BF_Provision-of-instructions	0.208	OS_Provision-of-instructions	0.000
BF_Self-appraisal	0.043	OS_Self-appraisal	0.004
BF_Social-support	0.022	OS_Social-support	0.019
DS_Behavior-self-monitoring	0.181	REMS_Behavior-self-monitoring	0.028
DS_Gamification	0.008	REMS_Gamification	0.007
DS_Goal-setting	0.735	REMS_Goal-setting	0.063
DS_Induction	0.071	REMS_Induction	0.189
DS_Personalization	0.214	REMS_Personalization	0.016
DS_Provision-of-instructions	0.024	REMS_Provision-of-instructions	0.042
DS_Self-appraisal	0.030	REMS_Self-appraisal	0.044
DS_Social-support	0.033	REMS_Social-support	0.047
HR_Behavior-self-monitoring	0.493	T_Behavior-self-monitoring	0.001
HR_Gamification	0.148	T_Gamification	0.002
HR_Goal-setting	0.562	T_Goal-setting	0.001
HR_Induction	0.035	T_Induction	0.000
HR_Personalization	0.007	T_Personalization	0.007
HR_Provision-of-instructions	0.185	T_Provision-of-instructions	0.016
HR_Self-appraisal	0.706	T_Self-appraisal	0.294
HR_Social-support	0.292	T_Social-support	0.133

In Table 5 it is possible to see that one or more sets of measures per vital sign don’t follow the normal distribution, for this reason, the Friedman test was used to verify the existence of significant differences in the impact of the behavior change technique per vital sign. The Friedman test hypothesis H0 establishes that there aren’t significant differences in a set of different related samples. If the p-value is lower than 0.05 the hypothesis H0 is annulled with a 95% of confidence interval. Table 6 shows the Friedman tests results where it is possible to see that there are significant differences between the sets of measures of each of the six vital signs analyzed. Table 7 shows the vital signs with statistically significant differences in the post-hoc Wilcoxon tests. Also, Table 8 shows the ranking of the behavior change technique from the better to worse impact well-being of health, taking into account the average range resulting from the Friedman test. The average range is a measure that indicates how the data of one group are distributed in relation to the other groups in a dataset, a high average rank compared to a low average rank of another data group suggests a significant difference. The post-hoc tests confirm the correct ranking of the made with the Friedman test average ranges excepts by the order of Provision-of-instruction and Self-appraisal change techniques. The Wilcoxon tests show that Self-appraised has more statistically significant differences in health well-being than Provision-of-instructions.

**Table 6:** Results of the Friedman tests per vital sing

	BF	DS	HR	OS	REMS	T
**N**	30	30	30	30	30	30
**Chi-square**	26.883	43.474	34.538	70.931	99.556	41.109
**D.F.**	7	7	7	7	7	7
**P-value**	0.000	0.000	0.000	0.000	0.000	0.000

**Table 7:** Vital signs with statistically significant differences in post-hocs Wilcoxon tests. Intersecting the behavior change techniques from a row to a column, the underlined vital signs represent that the BCT from the row is higher than the BCT from the column. Otherwise with vital signs not underlined

	Behavior-self-monitoring	Gamification	Goal-setting	Induction	Personalization	Provision-of-instructions	Self-appraisal	Social-support
**Behavior-self-monitoring**		HR, REMS	BF, DS, OS, REMS, T	BF, OS, REMS	HR, REMS	DS, OS, REMS, T	BF, OS, REMS, T	HR, OS
**Gamification**			BF, DS, HR, OS, REMS, T	BF, DS, OS, REMS, T	HR, REMS, T	DS, HR, OS, REMS, T	BF, DS, HR, OS, REMS, T	OS, REMS
**Goal-setting**				HR	BF, DS, OS, T			DS, HR, OS, REMS, T
**Induction**					BF, HR, OS	HR	REMS	DS, OS, REMS, T
**Personalization**						DS, OS	BF, DS, OS	HR, OS, REMS, T
**Provision-of-instructions**							BF	DS, HR, OS, REMS, T
**Self-appraisal**								DS, HR, OS, REMS, T

**Table 8:** Ranking of behavior change techniques from best to worst impact on vital signs based on average ranges of the Friedman test

Technique	BF	DS	HR	OS	REMS	T	Rank
Goal-setting	3.68	5.87	3.42	5.85	5.32	3.48	1
Provision-of-instructions	4.5	5.7	3.95	5.83	6.15	3.17	2
Self-appraisal	3.28	5.12	4.02	5.77	6.12	3.65	3
Induction	3.78	5.22	5.13	5.4	5.13	4.13	4
Personalization	5.23	3.68	3.35	3.62	5.2	4.58	5
Behavior-self-monitoring	5.5	3.85	4.53	3.62	2.67	5.43	6
Gamification	5.45	3.62	5.67	3.65	3.78	6	7
Social-support	4.57	2.95	5.93	2.27	1.63	5.55	8

**Fig. 5:**
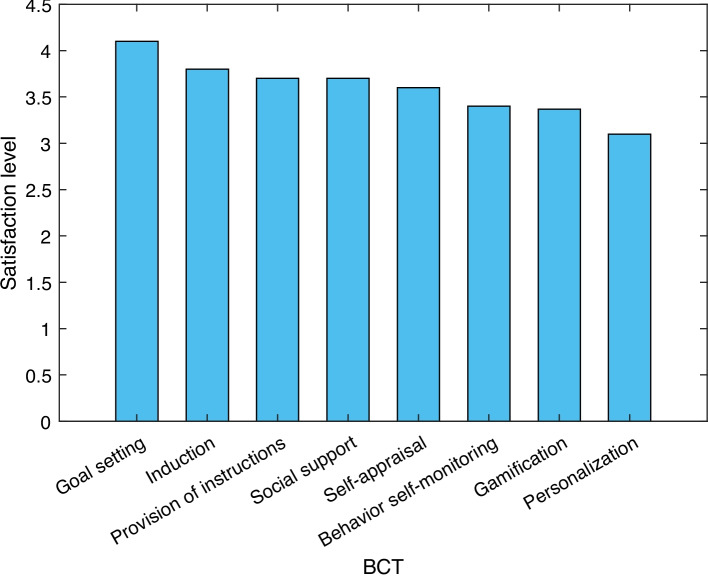
Level of average satisfaction by behavioral change techniques activities

In Fig. 5 is possible to see the general results of the satisfaction experienced by the samples in the behavior change techniques activities. The level of satisfaction was rated on a scale of 1 to 5, with 1 being the lowest level and 5 being the highest level of satisfaction. Also, Table 9 shows the rank of activities with the best to the worst rated satisfaction.

**Table 9:** Activities rank by satisfaction level

Activity	Level	Behavior change technique
Time for shorter routes	4.9	Goal setting
Daily activity notes	4.8	Self-appraisal
Mark number of steps	4.7	Goal setting
Phone reminders	4.7	Induction
Social network to share experiences and strategies	4.7	Social support
Classification of profiles to engage in personal communication	4.6	Personalization
Interactive online community	4.6	Social support
Social rank expectation and confirmation	4.5	Personalization
Total burned calories	4.2	Goal setting
Smart alert system	4.2	Induction
Provide graphs and messages with feedback tailored to progress	4	Behavior self-monitoring
Check-in places to have a record of favorites	4	Induction
Detailed instructions of tasks that can be performed to reduce sedentary behavior	4	Provision of instructions
Sleep activity log	4	Self-appraisal
Make shopping list	3.9	Provision of instructions
Auditory and visual route tracking	3.9	Provision of instructions
Collective challenges without borders	3.8	Gamification
Measurement of levels of concentration, stress and attention	3.7	Behavior self-monitoring
Monitoring of healthy lifestyles	3.7	Goal setting
Visit patterns for marketing quality	3.5	Social support
Earn points for every n steps	3.4	Gamification
Reinforcement learning	3.3	Behavior self-monitoring
Participatory learning	3.3	Behavior self-monitoring
Breathing exercises	3.3	Provision of instructions
Prioritizing the use of applications for productivity	3.3	Provision of instructions
Analysis of physical complexion	3.3	Self-appraisal
Fitness Monitoring Cycle	3.2	Gamification
Systems that include immersive video games	3.2	Gamification
Advanced dialog interfaces	3.2	Gamification
Daily food notes	3.2	Self-appraisal
Reminder for shopping list	3.1	Induction
Help people with disabilities in real time	3.1	Social support
Reminders to improve sleep behavior	3	Induction
Portable data monitoring	2.8	Behavior self-monitoring
Ideal weight monitoring	2.7	Goal setting
Monitoring of periodic physiological functions	2.6	Self-appraisal
Gesture control	2.6	Social support
Send personalized messages	2.3	Personalization
Position and activity data may recommend fluid intake	2.3	Personalization
Position and search data to send advertising and promotions	1.8	Personalization

## Discussion

The segmentation of data by continent provides an interesting global perspective on the use of wearables. The preference for certain types of wearables in different regions is a noteworthy finding, with Smart Watches being prominent in Latin America, Europe, and Asia. This suggests that cultural and regional factors may influence wearable technology adoption.

Figures 2 and 3 provide valuable insights into users’ perspectives on the advantages and disadvantages of wearables. The emphasis on quality of life improvement and data analytics as advantages aligns with the growing interest in self-tracking and health monitoring. Privacy and security concerns, as mentioned in the disadvantages, are critical issues that need to be addressed as wearables become more integrated into daily life.

In the experiment where we measured the impact of the wearable activities by behavior change technique, the satisfaction survey and the analysis of the vital signs match in the following cases:

The better level of satisfaction recorded and the better impact on the vital signs were observed in the Goal setting behavior change technique. Wearables and behavioral change techniques represent a powerful combination for promoting health and well-being. These devices, equipped with advanced sensors and integrated with BCTs, enable users to monitor vital signs and adopt healthier behaviors.

An interesting event happened with the social support activities, the persons rated this BCT with a good score in the satisfaction survey but the collected data show that is the worst BCT taking into account the well-being of the vital signs impact.

Under the theory of technological devices adapted to improving daily life and optimizing behavioral change techniques, we found some features:

- Self-Monitoring: our work evaluates the impact of wearables on behavior change, which often includes self-monitoring of physical activity. The CALO-RE Taxonomy of Behavior Change Techniques [46] explicitly mentions Self-monitoring of behavior and Self-monitoring of behavioral outcome as behavior-change techniques. Wearables can facilitate self-monitoring by providing real-time data on physical activity. This feature exhibits pronounced fluctuations with respect to the Deep Sleep metric and also demonstrates the highest values with regard to the temperature metric. This can lead to an analysis of the individual being more alert due to physical activity and certain challenges in daily activities, potentially resulting in some sleep disruption. Action planning involves creating detailed plans for when and where to engage in a behavior. This aligns with the idea of wearables providing real-time monitoring and feedback, as wearables can help users plan their physical activity and remind them when to act.
- Providing Feedback: Our work discusses Provide feedback on performance, while the The CALO-RE Taxonomy of Behavior Change Techniques [46] emphasizes the importance of feedback in promoting exercise. Wearables often offer immediate feedback on an individual’s physical activity performance. With regard to the oxygen saturation metric, the technique associated with goals and feedback exhibits the highest oxygenation average. Conversely, concerning Heart Rate, it displays the least accelerated levels, indicating a positive influence on health. In the realm of behavioral change techniques promoted by technological devices and wearables, the aforementioned metrics play a pivotal role. The oxygen saturation metric, particularly when coupled with goal-setting and real-time feedback mechanisms, showcases the most promising results by consistently maintaining higher oxygenation averages. This not only encourages individuals to strive for better health outcomes but also provides them with actionable insights to make informed choices. On the other hand, the Heart Rate metric, with its ability to maintain lower and stable levels, suggests a positive impact on overall health. Wearables equipped with such data-driven capabilities not only monitor but also actively contribute to individuals’ well-being by fostering a heightened awareness of their physiological responses, thus facilitating meaningful behavioral changes for the better.
- Information Provision: our work discusses how wearables provide information about users’ health and behavior, while the CALO-RE Taxonomy of Behavior Change Techniques [46] mentions Information provision (general) and Information provision (to the individual) as techniques. Wearables provide users with information about their activity levels, encouraging awareness and behavior change. Regarding the provision of instructions, the REM sleep metric and the temperature metric both exhibit a state of heightened relaxation for the individual. These wearables offer invaluable insights into various aspects of an individual’s well-being. For instance, in terms of providing instructions for better sleep quality, wearables have demonstrated a positive impact. Metrics like REM sleep and temperature regulation play pivotal roles in achieving a relaxed state during sleep. The REM sleep metric, in particular, highlights periods of deep restorative sleep, while the temperature metric reflects the body’s ability to create a conducive sleep environment. These insights empower individuals to make informed choices about their sleep habits, contributing positively to their overall health. By fostering awareness and facilitating behavioral adjustments, wearable devices have the potential to enhance various vital signs, ultimately leading to improved health outcomes.

This research is commendable for its comprehensive approach to studying behavior change techniques and their effects on individuals’ well-being. By using wearables and monitoring vital signs, the study aims to provide objective data on the impact of these techniques, which can be crucial for evidence-based interventions and recommendations. One strength of the study is its clear and well-structured methodology. The division of participants into groups exposed to different behavior change techniques for specific durations allows for controlled testing of each technique’s effectiveness. Additionally, the use of satisfaction surveys provides valuable subjective insights into participants’ experiences.

The present work appropriately discusses the statistical methods employed for data analysis, including tests for normality, ANOVA, and Friedman tests. These tests are commonly used in research to determine the significance of differences between groups. However, the text does not specify the significance level (alpha) used for these tests, which is essential information for interpreting the results.

Furthermore, the inclusion of a detailed table (Table 2) that categorizes behavior change techniques and their corresponding activities is a helpful reference for readers. It provides transparency about the specific techniques tested in the study, helping readers understand the scope of the research.

Goal setting, personalization, and gamification are among the most effective BCTs, positively impacting user satisfaction and vital signs. The integration of wearables with digital platforms and online communities further enhances their potential.

However, privacy and security concerns must be carefully addressed to maintain user trust and engagement. As technology continues to evolve, wearables have the potential to revolutionize health monitoring and behavior change, offering users valuable insights and personalized interventions for improved well-being.

## Conclusion

Big data analytics aims to identify trends and patterns that can be used to make decisions, predict behavior, or create machine learning and artificial intelligence models. Thanks to advances in mobile technology, it is possible to insert sensors into devices to locate their users and capture their movements, emotions, and degree of social bonding. This facilitates the application of self-regulatory techniques, such as goal setting and monitoring. In addition, connectivity allows data on habits and health to be shared with health personnel or other people, facilitating behavioral change. The impact of long-term behavioral change efficacy is an interesting topic that could be future work. In this work, the main applications are analyzed based on the average wearable devices in the continents, according to the needs and profiles of users. We found the highest value in Fitness trackers and health gadgets. In addition, we give a biometric overview of the use of these devices according to the predilection of people in their daily lives. We make quantifications of all the advantages and disadvantages found by users of wearables worldwide, finding that the most significant are Quality of life and Privacy and security, respectively. Finally, we do a change analysis of wearable-enhanced behavioral techniques and experiment with activities for each technique to observe the behavior of users with and without wearables. We found that some behavioral change techniques are not perceived as modified by wearables, such as social support and provisions of instructions. The other techniques, such as Induction, Goal setting, Gamification, Personalization, and Behavior self-monitoring, are perceived positively when people use wearables and negatively when they do not. These devices can be essential for changing lifestyles; for example, they effectively address physical inactivity, social interaction, and sedentary behavior. Similarly, they can help people take more active control of their health and promote empowerment. However, there is still resistance to implementing applications related to wearables due to the still-existing gap of technological appropriation in some countries of the world.

### Supplementary Information

Supplementary Material 1.

## Data Availability

Data Availability can be sent to the Journal through the Supplementary Material.
